# Tolerance of repeated toxic injuries of murine livers is associated with steatosis and inflammation

**DOI:** 10.1038/s41419-023-05855-4

**Published:** 2023-07-12

**Authors:** Seddik Hammad, Christoph Ogris, Amnah Othman, Pia Erdoesi, Wolfgang Schmidt-Heck, Ina Biermayer, Barbara Helm, Yan Gao, Weronika Piorońska, Christian H. Holland, Lorenza A. D’Alessandro, Carolina de la Torre, Carsten Sticht, Sherin Al Aoua, Fabian J. Theis, Heike Bantel, Matthias P. Ebert, Ursula Klingmüller, Jan G. Hengstler, Steven Dooley, Nikola S. Mueller

**Affiliations:** 1 Molecular Hepatology Section, Department of Medicine II, University Medical Center Mannheim, Medical Faculty Mannheim, Heidelberg University, Mannheim, Germany; 2 Department of Forensic Medicine and Veterinary Toxicology, Faculty of Veterinary Medicine, South Valley University, Qena, Egypt; 3 Institute of Computational Biology, Helmholtz-Zentrum München, German Research Center for Environmental Health, Neuherberg, Germany; 4 Department of Toxicology, Leibniz Research Centre for Working Environment and Human Factors (IfADo), Dortmund, Germany; 5 Leibniz Institute for Natural Product Research and Infection Biology - Hans Knoell Institute, Jena, Germany; 6 Division Systems Biology of Signal Transduction, German Cancer Research Center (DKFZ), INF 280, Heidelberg, Germany; 7 Core Facility Next Generation Sequencing, Medical Faculty Mannheim, Heidelberg University, Mannheim, Germany; 8 Department of Gastroenterology, Hepatology and Endocrinology, Hannover Medical School, Carl-Neuberg-Strasse 1, Hannover, Germany; 9 Department of Medicine II, University Medical Center Mannheim, Medical Faculty Mannheim, Heidelberg University, Mannheim, Germany; 10 Mannheim Institute for Innate Immunoscience (MI3), University Medical Center Mannheim, Medical Faculty Mannheim, Heidelberg University, Mannheim, Germany; 11 Clinical Cooperation Unit Healthy Metabolism, Center of Preventive Medicine and Digital Health, University Medical Center Mannheim, Medical Faculty Mannheim, Heidelberg University, Mannheim, Germany

**Keywords:** Liver fibrosis, Prognostic markers, Experimental models of disease, Transcriptomics

## Abstract

The human liver has a remarkable capacity to regenerate and thus compensate over decades for fibrosis caused by toxic chemicals, drugs, alcohol, or malnutrition. To date, no protective mechanisms have been identified that help the liver tolerate these repeated injuries. In this study, we revealed dysregulation of lipid metabolism and mild inflammation as protective mechanisms by studying longitudinal multi-omic measurements of liver fibrosis induced by repeated CCl_4_ injections in mice (*n* = 45). Based on comprehensive proteomics, transcriptomics, blood- and tissue-level profiling, we uncovered three phases of early disease development—initiation, progression, and tolerance. Using novel multi-omic network analysis, we identified multi-level mechanisms that are significantly dysregulated in the injury-tolerant response. Public data analysis shows that these profiles are altered in human liver diseases, including fibrosis and early cirrhosis stages. Our findings mark the beginning of the tolerance phase as the critical switching point in liver response to repetitive toxic doses. After fostering extracellular matrix accumulation as an acute response, we observe a deposition of tiny lipid droplets in hepatocytes only in the Tolerant phase. Our comprehensive study shows that lipid metabolism and mild inflammation may serve as biomarkers and are putative functional requirements to resist further disease progression.

## Introduction

Chronic liver injury causes ongoing cell death of liver cells, resulting in a gradual replacement of the normal liver with fibrotic tissue, leading to cirrhosis in a long-term process [1, 2]. It triggers a complex response cascade characterized by infiltration and activation of the immune cells in the liver, leading to both inflammatory and wound-healing responses and fibrogenesis [3, 4]. In addition, hepatic stellate cells (HSC) change to a myofibroblast phenotype characterized by (i) high proliferative activity; (ii) expression of extracellular matrix (ECM) components; (iii) gain of contractility, chemotaxis, and migratory properties; and (iv) the production of large amounts of growth factors and profibrogenic cytokines promoting fibrogenesis, e.g., TGF-β [5, 6]. Maintaining the balance between quiescence and activation of HSC is a highly dynamic and convoluted process [7–9]. Accordingly, liver disease progression and regression are highly variable and can take many years to develop progressed fibrosis/cirrhosis at the patient’s level [1, 10, 11]. Fortunately, liver fibrosis and even early cirrhosis are reversible, as shown previously in experimental and clinical settings [12].

Previous studies on liver fibrosis in response to toxic injury primarily compared stable disease states, like resistant versus susceptible mouse strains, including four or six weeks of repetitive toxic exposure, e.g., carbon tetrachloride (CCl_4_). Recently, Tuominen et al. [13] examined the susceptibility of 98 mouse strains (693 livers) to six weeks of fibrosis-inducing CCl_4_ administration using transcriptomics. Their report assigned the top 300 down-regulated genes to metabolic pathways, e.g., biological oxidations, steroids, and lipids [13]. In addition, several studies analyzed the liver responses to CCl_4_ after four to six weeks of administration [7, 14–16]. However, dynamic multi-level liver response changes to repeated injuries and the critical time point in switching response mechanisms have yet not been identified.

Our study shines a light on these knowledge gaps via longitudinal multi-omic measurements of liver fibrosis-induced mice. We established a mouse model with repetitive CCl_4_ injections twice a week, for ten weeks. We collected samples (*n* = 3–6) each week using oil-treated mice (*n* = 6) in weeks zero and ten as control. Bi-weekly profiling of proteomics, transcriptomics, and weekly blood- and tissue-level profiling delineated three independent phases of liver responses: Initiation phase as immediate response patterns; Progression phase of accumulating molecular changes; Tolerance phase to endure repeated injury. To identify critical regulatory liver processes that dampen the effect of fibrosis, we focused our analysis on the Tolerance phase. In detail, we used a novel multi-omic network-based data integration strategy [17] to reveal 13 tolerance-specific key mechanisms, including lipid metabolism and mild inflammation. A comprehensive histological and biochemical validation proved an increase in the accumulation of tiny intracellular lipid droplets and mild inflammation within the fibrotic tissue regions, especially during the tolerance phase independent of lipophagy. Moreover, a comparison to public datasets uncovered concordant regulation patterns of these dysregulated injury-tolerant processes in human liver diseases, including early fibrosis and compensated cirrhosis stages. Our findings led us to speculate whether these processes may act as autoprotective mechanisms to dampen the effects of fibrosis and represent potential markers for diagnosing the quiescent state of chronic liver diseases.

## Materials and methods

### Animal models of hepatic fibrosis

**Fig. 1:**
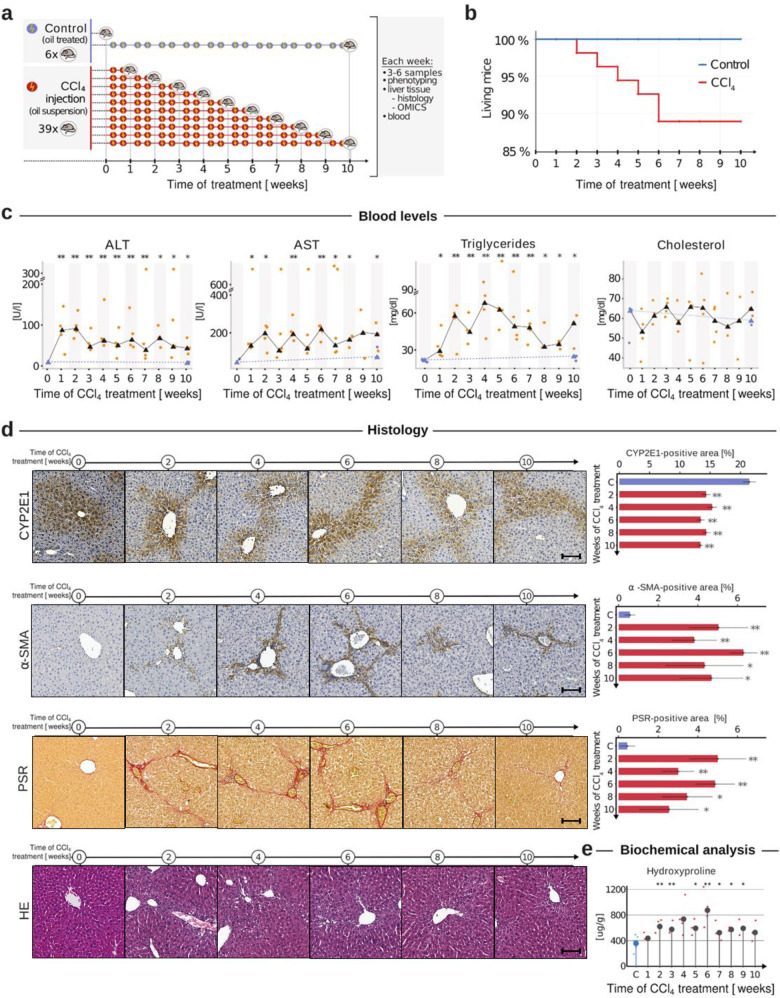
Longitudinal blood, histopathological and biochemical analysis of liver fibrosis dynamics. **a** Experimental setup using carbon tetrachloride (CCl_4_) administration in mice twice per week for 10 weeks. Blood and liver were collected weekly for further multi-level analysis. Oil-treated mice of weeks zero and ten were used as control. **b** Kaplan–Meier curve for survival analysis. **c** Longitudinal blood-based analysis of alanine aminotransferase (ALT), aspartate aminotransferase (AST), triglycerides, and cholesterol. Results are presented as the mean of 3–6 mice ± SD per week, and a significant difference to control is denoted via *p < 0.05, **p < 0.01. **d** Cytochrome P4502e1 (CYP2E1), alpha-smooth muscle actin (α-SMA), picro-sirius red (PSR), and hematoxylin & eosin (HE) staining with positive quantified signals as a percentage of total area. Scale bars are 100 µm. **e** Biochemical analysis of the Hydroxyproline level development over the ten-week treatment period.

Adult male C57Bl/6N mice were obtained from the Janvier Labs (France), housed three per cage in a temperature-controlled (24 °C) room with a 12-h light/dark cycle, and given ad libitum access to water and laboratory diet (Ssniff, Germany). Mice were maintained for seven days before carbon tetrachloride (CCl_4_) intoxication. The dose of CCl_4_ (Sigma-Aldrich, Cat. no. 319961) was 1.6 g/kg body weight [18] and was prepared as follows: to 3 ml of olive oil, 1 ml of CCl_4_ was added and mixed well. Mice received CCl_4_ intraperitoneally twice per week. In a time-resolved manner including weeks 1–10, mice were sacrificed at day two after the last CCl_4_ injection. An identical concentration of olive oil was injected into control groups for 10 weeks (Fig. 1a). At the indicated time point, blood and livers were harvested. The liver lobes were separated as follows: the caudate lobe (for hydroxyproline), right lobe (for proteomics and transcriptomics), and median lobe (cryosectioning) were freshly frozen in liquid nitrogen and stored at −80 °C. Further, left liver lobes were fixed in 4% paraformaldehyde (PFA) and embedded in paraffin for histopathological investigations. The experimental protocols with animals were carried out in full compliance with the guidelines for animal care and were approved by the Animal Care Committee from the German government (Animal permission number: 35.9185.81/G-216/16).

### STAM mouse model

STAM mouse (background C57BL6/j) livers were purchased from Stelic Institute and Co., Inc. (Japan). The mice were generated as described in [19], and lipid accumulation was quantified [20]. Cryosections from 8 weeks old mice (steatohepatitis stage) were prepared and stained with Bodipy as a positive control for comparison with CCl_4_-exposed mice.

### Clinical chemistry

Blood was collected in Li-Heparin vials from the retrobulbar plexus and centrifuged at 13,000 rpm at 4 °C for 6 min. Plasma was subsequently stored at − 80 °C until further analysis. Then, alanine aminotransferase (ALT), aspartate aminotransferase (AST), alkaline phosphatase (ALP), glucose (Gluc), triglycerides (TG), total protein (Prot), blood urea nitrogen (BUN) and cholesterol (Chol) were measured using a Hitachi automatic analyzer (Core facility-Medical Faculty Mannheim, Germany).

### RNA isolation and transcriptome analysis

Pieces of the right liver lobes were used to perform mRNA isolation with the InviTrap®Spin Universal RNA Mini Kit from Stratec (1060100300, Birkenfeld, Germany) according to the manufacturer. RNA concentration and integrity were summarized in Supplemental Table 1. Transcriptomics using the isolated mRNA from liver tissues (0, 2, 4, 6, 8, and 10 weeks; *n* = 3 per time point) was performed by Affymetrix GeneChip®Mouse Gene 2.0 ST Arrays (902118). Affymetrix-based transcriptomics was performed at NGS Core Facility, Medical Faculty of Mannheim, Germany (https://www.umm.uni-heidelberg.de/core-facilities/next-generation-sequencing/ngs/).

### Transcriptomic data preprocessing and bioinformatic analysis

Gene expression data obtained by the whole-transcript array GeneChip Mouse Gene 2.0 ST were pre-processed using ‘affyPLM’ packages of the Bioconductor Software [21]. Genes with the strongest evidence of differential expression were obtained using a linear model provided by the limma-package [22]. Data obtained from untreated mice were used as a reference. To annotate the microarrays, a custom chip definition file version 22 from Brainarray [23] based on Entrez IDs was used. A false-positive rate of α = 0.05 with false discovery rate (FDR) correction and a fold change greater than 1.5 was taken as the level of significance. To unravel patterns in the gene expression data for different pathways, the heatmaps ‘ComplexHeatmap’ [24] package was used. The raw and normalized gene expression profiling data have been deposited in NCBI’s Gene Expression Omnibus and are accessible through GEO Series accession number GSE222576 (https://www.ncbi.nlm.nih.gov/geo/query/acc.cgi?acc=GSE222576).

### Reverse transcription-polymerase chain reaction (RT-PCR)

Using RNA isolated for transcriptomics analysis, cDNA was produced from oligo(dT)18 primer (SO132, Thermo Scientific, Massachusetts, USA), dNTP Mix (R0191, Thermo Scientific Massachusetts, USA), and RevertAid H Minus Reverse Transcriptase (EP0451, Thermo Scientific, Massachusetts, USA), and then used for real-time (rt)-PCR (5× HOT FIREPol EvaGreen qPCR Mix Plus (ROX), 08-24-00020, Solis BioDyne, Tartu, Estonia) in a StepOne machine. Sequences of primer pairs were listed in Supplemental Table 2. All primers were purchased from Eurofins Genomics (Ebersberg, Germany). The mRNA expression levels of the detected genes were normalized to that of Ppia.

### Sample preparation for proteome analysis

The proteome profiling of liver tissue was performed at selected time points (0, 1, 2, 4, 6, 8, and 10 weeks; *n* = 3 per time point). Liver tissue was powdered using a Micro-Dismembrator (B.Braun, Micro-Dismembrator U Ball Mill), and approximately 10 mg aliquots of tissue powder were lysed in 100 µl SDS buffer (4% SDS, 1× Halt protein inhibitor, 40 mM TCEP, 160 mM CAA, 200 mM TEAB) by sonication on ice (60 s, 80% amplitude, 0.1 s off/0.5 s on) and centrifugation (15 min, 14,000 rpm, 4 °C). The supernatant was incubated at 95 °C for 5 min and 70 °C for 30 min for reduction and alkylation. Protein concentrations were determined by BCA assay, and 20 µg of total protein was used for subsequent tryptic digestion. Samples were prepared using a modified version of the Single-pot, solid-phase-enhanced sample preparation (SP3) protocol [25]. Briefly, a mix of Sera-Mag SP3 beads was added to the protein samples in a 10:1 SP3 beads/protein (wt/wt) ratio. Acetonitrile was added for a final concentration of 70% organic, and the mix was incubated for 18 min at room temperature (RT). Protein-bound beads were isolated on a magnetic rack and washed twice with 70% ethanol. A third wash was performed using 100% acetonitrile (ACN). Beads were air-dried and reconstituted in 100 mM TEAB buffer containing Trypsin Gold (Promega) in a 1:25 enzyme/protein (wt/wt) ratio. Protein digestion was performed for 14 h at 37 °C. The digested peptides were dried by vacuum centrifugation and stored at −20 °C until further use. A sample pool, consisting of 5 µg of each sample in the dataset, was generated. Peptides were then reconstituted in 50 mm HEPES (pH 8.5), and TMT10-plex reagents (ThermoFisher) were added to the samples (stocks dissolved in 100% ACN) in a 1:10 sample/TMT (wt/wt) ratio. The peptide–TMT mixture was incubated for 1 h at RT, and the labeling reaction was stopped by the addition of 5% hydroxylamine to a final concentration of 0.4%. Different samples were combined and the TMT-plexes were fractionated into 6 fractions using stage-tip Strong cation-exchange (SCX) fractionation. Stage-tips were manually prepared using 3 discs of SCX resin (Empore) and conditioned with MeOH, followed by 80% ACN, 0.5% AcOH; 0.5% AcOH; 500 mM NH4AcOH, 0.5% AcOH, 30% ACN; 20 mM NH4AcOH, 0.5% AcOH, 30% ACN, and was equilibrated with 0.5% AcOH successively. Before SCX fractionation, samples were reconstituted in 0.5% AcOH, sonicated, incubated on a shaker, and loaded onto the stage tips by centrifugation. Loaded stage tips were washed with 0.5% AcOH. For elution, descending concentrations of elution buffers (20/40/70/100/250/500 mM of NH4AcOH, 0.5% AcOH, 30% ACN) were used and the flow-through was collected. Fractions were dried by vacuum centrifugation and stored at −20 °C.

### LC-MS/MS Measurements

Nano-flow LC-MS/MS was performed by coupling an EASY-nLC (Thermo Scientific, USA) to a Q Exactive HF-X—Orbitrap mass spectrometer (Thermo Scientific, Germany). The fractions were dissolved in 11 µl loading buffer (0.1% formic acid, 2% ACN in LC-MS grade water), sonicated, and incubated on a shaker. 5 µl of each fraction was used for each measurement. Peptides were delivered to an analytical column (75 µm × 30 cm, packed in-house with Reprosil-Pur 120 C18-AQ, 1, 9 µm resin, Dr. Maisch, Ammerbuch, Germany) at a flow rate of 3 µl/min in 100% buffer A (0.1% formic acid in LC-MS grade water). After loading, peptides were separated using a 120 min stepped gradient from 6% to 50% of solvent B (0.1% formic acid, 80% ACN in LC-MS grade water; solvent A: 0.1% formic acid in LC-MS grade water) at 350 nL/min flow rate. The Q Exactive HF-X was operated in data-dependent mode (DDA), automatically switching between MS and MS2. Full-scan MS spectra were acquired in the Orbitrap at 120,000 (m/z 200) resolution after accumulation to a target value of 3,000,000. Tandem mass spectra were generated for up to 18 peptide precursors in the Orbitrap (isolation window 0.8 m/z) for fragmentation using higher-energy collisional dissociation (HCD) at a normalized collision energy of 32% and a resolution of 45,000 with a target value of 50,000 charges after accumulation for a maximum of 96 ms.

### Protein identification, quantification, and statistical analysis

Raw MS spectra were processed by MaxQuant (version 1.6.0.1) for peak detection and quantification. MS/MS spectra were searched against the Uniprot mus musculus reference proteome database (downloaded on October 22nd, 2018) by the Andromeda search engine enabling contaminants and the reversed versions of all sequences with the following search parameters: Carbamidomethylation of cysteine residues as fixed modification and Acetyl (Protein N-term), Oxidation (M) as variable modifications. Trypsin/P was specified as the proteolytic enzyme with up to 3 missed cleavages allowed. The mass accuracy of the precursor ions was decided by the time-dependent recalibration algorithm of MaxQuant. The maximum false discovery rate (FDR) for proteins and peptides was α = 0.01 and a minimum peptide length of eight amino acids was required. Quantification mode with isobaric labels (TMT 10plex) was selected. All other parameters are defined as default settings in MaxQuant.

### Proteomic data preprocessing and bioinformatic analysis

By filtering out contaminant proteins, as indicated by MaxQuant analysis, and using the corrected reporter intensity for analysis, all proteins with at least one missing expression value in one of the samples were removed, leaving 2278 proteins for subsequent analysis. This stringent cutoff was chosen as visual inspection of the data showed that missingness increases with reporter intensity, thus protein expression. We computed the ratio per p protein j on the basis of its sample’s expression s (pj,s) and the samples-matching protein expression in the TMT10plex isotope reference channel r (rj) according to (1+pj,s)/(1+rj). One outlier sample was removed after this stage. Robust quantile normalization was applied to the data using the MSnSet R package, resulting in final normalized expression ratios used for statistical analysis. For the identification of differentially regulated proteins per time point (1, 2, 4, 6, 8, and 10 weeks) when compared to the control time point (0 weeks), we used a multivariate linear model with time-point specific dummy variables and protein ratio as a response. To call differential expression, we subjected p-values of all proteins separately per coefficient to multiple testing corrections with the Benjamini Hochberg procedure, also referred to as False-Discovery Rate. The proteome data is uploaded to the Proteome Xchange Consortium via PRIDE (Access number: PXD030956). Differential expression per time-point was set to FDR below α = 0.05.

### Multi-omics network inference and analysis

The multi-omic network inference was performed using KiMONo [17]. This novel versatile tool can use any kind and any amount of omic data by leveraging prior knowledge. By doing so KiMONo generates a multi-level network around an omic type of interest, simplifying downstream analysis, i.e., pathway analysis. In the final multi-level network, nodes represent features like proteins, genes, or clinical variables and the connections between them denote effects identified within the input data. Here, we used KiMONo to generate three networks centered around the three given data types and combined them to enhance the signal within the time-resolved data. This was done by only reporting effects that were found in all three multi-omic networks. To infer each network, we used three different priors providing information about already known relations between the transcriptomic, proteomic, and clinical data. In KiMONo the priors serve as a rough blueprint, reducing the complexity and improving the algorithm’s performance. The first priority was obtained via the Biomart tool annotating genes to proteins. The second priority is based on the BioGRID database to include information about protein-protein relations [26]. As a third priority, we used all previous annotations to identify indirect gene-protein interactions. This was done by using BioGRID interactions information also to annotate genes to proteins of their coding protein. Finally, we set off to generate a prior, which would annotate the omic information to our clinical data. Therefore, we first inferred the transcriptomic and proteomic centered networks and used the information about all potential effects of clinical features prior to the clinical centered network inference. The importance of network nodes was estimated via the network’s betweenness centrality. This measure is estimated by determining the shortest path between all nodes within the graph. The betweenness centrality for a node is then estimated by the number of shortest paths that pass through a node. Small modules (2–3 nodes) were manually functionally annotated to biological function using the GeneCards database [27], avoiding false positives during the annotation process. All other modules were first annotated using the online pathway analysis tool PathwaX II [28] and the KEGG [29] and Reactome [30] pathways. Significantly, enriched pathways (FDR < 0.05) were manually curated to identify overarching functional themes which were assigned as labels to modules. Moreover, we also manually annotated small modules (2–3 elements) via literature search.

### Comparative analysis with available mouse and human cohorts

We obtained 1034 human samples across 11 liver disease-related datasets (different etiologies) via NCBI’s Gene Expression Omnibus (GEO), see Supplemental Table 3. Significant differentially expressed (DE) genes (FDR < 0.1) have been extracted by using the GEO2R interface and its default settings. Further, we identified mouse orthologous genes using the Inparanoid 8 databases [31]. For further validation, we analysed the identified 210 *tolerance* genes with liver expression data of a human cohort of different stages of chronic liver diseases (GSE139602) including early fibrosis and compensated cirrhosis as well as mice of different ages upon long-term CCl_4_ treatment (GSE167216).

### Hepatic hydroxyproline determination

Hydroxyproline (HYP) was determined colorimetrically in triplicates from snap-frozen liver lobes as described in Fels [32] with modifications. Briefly, approximately 100 mg of tissue from the caudate liver lobe was homogenized and hydrolyzed in 2 ml of 6 N HCl at 110 °C for 16 h. HYP content was then measured photometrically at 558 nm. Based on relative HYP (per 100 mg of the frozen liver), total hepatic HYP was calculated (total liver, as obtained by multiplying liver weights with relative hepatic HYP).

### Liver histology and Immunohistochemistry

The left lobe was fixed in 5 ml 4% PFA at 4 °C for 2 days for paraffin embedding. Formalin-fixed, paraffin-embedded (FFPE) liver sections were stained with hematoxylin and eosin (H&E) for assessment of liver structures and inflammation. For assessment of hepatic fibrosis, FFPE sections were stained with Sirius Red (Sigma, 365548-5 G). FFPE liver sections were incubated with primary antibodies against α-smooth muscle actin (α-SMA) (Abcam, ab5694, 1:100), rat anti-F4/80 (BioRad, MCA497R, 1:100), or rabbit anti-CYP2E1 (Sigma, HPA009128, 1:100) to assess activated HSC, resident macrophages, and pericentral hepatocytes, respectively. The slides were scanned shortly after the staining procedure using the slide scanner Aperio 8 (Leica). Digital pathological analysis was performed using ImageJ (https://imagej.nih.gov/ij/) on an equal number of pictures per mouse (10–15 images) under constant magnification (10X).

### Preparation of cryosections, staining of lipid droplet and autophagy and lipophagy markers

Part of the median lobe was embedded in tissue-Tek (VWR, 25608-930) and kept at −80 °C till cryosectioning. Cryosections (5μm thickness) were fixed in 4% PFA for 15 min, then briefly washed with running tap water and 60% isopropanol. Then cryosections were incubated with Bodipy (Life Technologies, D-3922, 1:250), LC3 (Abgent, AP1802a; 1:100), LAMP1 (Abcam, ab24170, 1:100), or PLIN3 (ProSci, 3883, 1:100) for 30 min. After rinsing steps with 60% isopropanol, cryosections were incubated for 30 min with donkey anti-rabbit cy3 (Diannova, 711-166-152, 1:200). Finally, the slides were incubated either with Draq5 (Cell Signaling Technology, 4084 L, 1:5000) or DAPI (Invitrogen, D1306, 1:1000). Two tile scans of 9 images each per mouse for quantification were acquired using confocal microscopy (Leica SP8, UMM-Core facility Mannheim, Germany). Lipid droplets quantification was performed using ImageJ (https://imagej.nih.gov/ij/) on tile scans.

### Statistical analyses

Statistical analyses and heatmaps were performed in Prism (Version 8, GraphPad Software). Data are shown as mean ± SD of 3–6 mice per group and the two-tailed Student’s t-test was calculated when shown. *p*-values < 0.05 (*), < 0.01 (**), < 0.001 (***), < 0.0001 (****) are indicated.

## Results

### Liver response program switches after six weeks of induced fibrosis

Liver fibrosis is induced by repetitive injections of CCl_4_ for 10 weeks (Fig. 1a). The survival analysis shows that within the first six weeks each week, 5% of CCl4-administered mice died, resulting in a total loss of 10% of the tested animals. Interestingly, the repeated injuries caused no further mortality after week six (Fig. 1b), suggesting that post-six-week survivors developed an injury-tolerant response program. During the same treatment period (weeks 0–6), all mice treated with CCl_4_ had a significant liver-to-body weight gain (Supplemental Fig. 1a and b). Moreover, we observed that the alterations in survival rate were accompanied by an oscillating increase of well-established liver damage biomarkers—serum alanine aminotransferase, ALT (*p* < 0.05), and aspartate-transaminase, AST (Fig. 1c) (*p* < 0.05). ALT levels peaked after two weeks of treatment, while we detected no further increase of AST after week six. Markers for bile duct damage (alkaline phosphatase, ALP) and kidney function (blood urea nitrogen, BUN) showed no significant alterations compared to the control (Supplemental Fig. 1c, d). Blood triglyceride (TG) levels presented a continuous increase in a time-dependent manner until week five (Fig. 1c) and returned to control levels towards week ten.

Additionally, we observed a trend for a decrease in glucose and total protein content (Supplemental Fig. 1f) but no significant changes in blood cholesterol (Chol) (Fig. 1c). These blood level analysis findings represent a first response to the CCl_4_-induced fibrosis within weeks zero to six. Combined with the phenotypic information and the survival rate, it suggests a switch of the liver response program between weeks five and seven to a tolerable fibrosis phase.

### The longitudinal tissue-based analysis suggests three liver response phases

We performed a comprehensive histological and biochemical analysis to examine the liver response dynamics on a structural level. We determined the protein level (Fig. 1d, CYP2E1) and mRNA expression of Cytochrome P4502e1 (CYP2E1), the key metabolizing enzyme of CCl_4_, using RT-PCR (Supplemental Fig. 1g). Both mRNA and protein levels showed constant levels of CYP2E1 even after week six of repeated liver injury, indicating a stable and lower metabolic capacity of the liver. Quantitative morphometric assessment of alpha-smooth muscle actin (α-SMA positive), representing activated hepatic stellate cells (HSC), revealed massive accumulation during the first six-week period of repeated CCl_4_ exposure (Fig. 1d, α-SMA). Despite the continuous CCl_4_ administration, no further increase in α-SMA positivity was observed at the later time points (Fig. 1d, α-SMA positive area). Likewise, the ECM deposition analyzed by quantifying picrosirius red (PSR) positive areas showed the same behavior and did not increase beyond six weeks of exposure (Fig. 1d, PSR). Using a biochemical assay, we evaluated the hydroxyproline liver levels, HYP, a major component of collagen, to show that the ECM-related changes are not local or sparse events but organ-wide alterations caused by continuous CCl_4_ administration (Fig. 1e). Finally, H&E staining demonstrated marked cell necrosis, inflammatory reaction, and formation of septal damage (Fig. 1d, HE). The significant non-linear fibrotic tissue accumulation corresponds to METAVIR stage 2 or 3 of fibrogenesis. Hence, this result aligns with a previous report showing an increase in METAVIR stage is associated with a progressive non-linear increase in the fibrosis area [10]. This biochemical and histopathological analysis underpins the notion of different response phases. Moreover, the findings show a response phase that tolerates repeated injuries after six weeks, independent of the metabolic bioactivation of CCl_4_.

### Transcriptomics and proteomics divide the identified regulatory programs of liver fibrosis into the initiation, progression, and tolerance phase

**Fig. 2:**
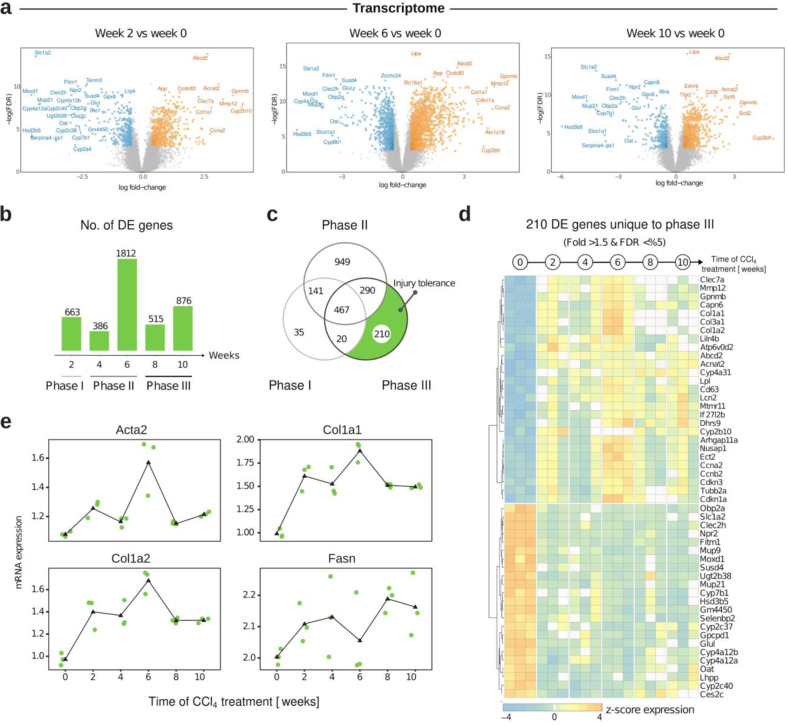
Time-resolved transcriptome analysis of liver exposed to CCl_4_. **a** Volcano plots illustrate identified DE genes of weeks two, six, and ten of CCl_4_ exposure. **b** The number of DE genes is visualized in the bar chart. Time points are grouped into three phases characterized by the disease dynamics of liver fibrosis. **c** Venn diagrams show the unique and shared amount of genes between the phases. **d** Heatmap of 210 phase III specific DE genes (*n* = 3 mice per time point). **e** Diagram visualizing the regulation in a time-resolved manner of the genes; Acta2, Col1a1, Col1a2, and Fasn.

The dynamics of structural changes detected at the tissue level indicate underlying molecular alteration best studied at the transcriptional and proteomic levels. Therefore, we profiled the transcriptome of 18 mice across weeks two, four, six, eight, and ten using Affymetrix microarrays (Supplemental Table 1) and RT-PCR for several fibrogenic genes (Supplemental Fig. 2). Differentially expressed (DE) genes between control and two, six, and ten weeks were identified using an FDR < 0.05 and absolute log fold change > 1.5 (Fig. 2a). The highest number of 1812 DE genes was observed at week six (Fig. 2b, c), supporting the tissue-level analysis (We also detected the highest structural alteration during the sixth week). Based on a Principal Component Analysis (PCA), we grouped the time points shaping three phases of early liver fibrosis—phase I (week zero–three), phase II (week four–six), and phase III (week seven–ten) (Supplemental Fig. 3a). Pathway annotation of each phase’s DE geneset identified several metabolic pathways and the ECM pathway as enriched (Supplemental Fig. 3a). These are activated throughout phases I & II and stabilized or reduced during the last phase III (Supplemental Fig. 3). Between these DE sets, 467 genes were commonly regulated and enriched for ECM and inflammation pathways (Fig. 2c). We identified reduced molecular regulation (210 unique DE genes, Fig. 2d) in phase III, reflecting previous findings and marking an adaptation to repeated injuries; hence we coined phase III—the tolerance phase. Analysis of time-resolved expression of the tolerant DE genes (Fig. 2e) underlines the dynamics of fibrogenic genes, e.g., Actin alpha 2, smooth muscle (Acta2), Collagen type I alpha 1 chain (Col1a1), and Collagen type I alpha 2 chain (Col1a2), until week six and a decreased regulation during the tolerance phase. Additionally, we can observe an increase in fatty acid synthase (Fasn) (lipogenic gene) during the late tolerance state (Fig. 2d, e).

**Fig. 3:**
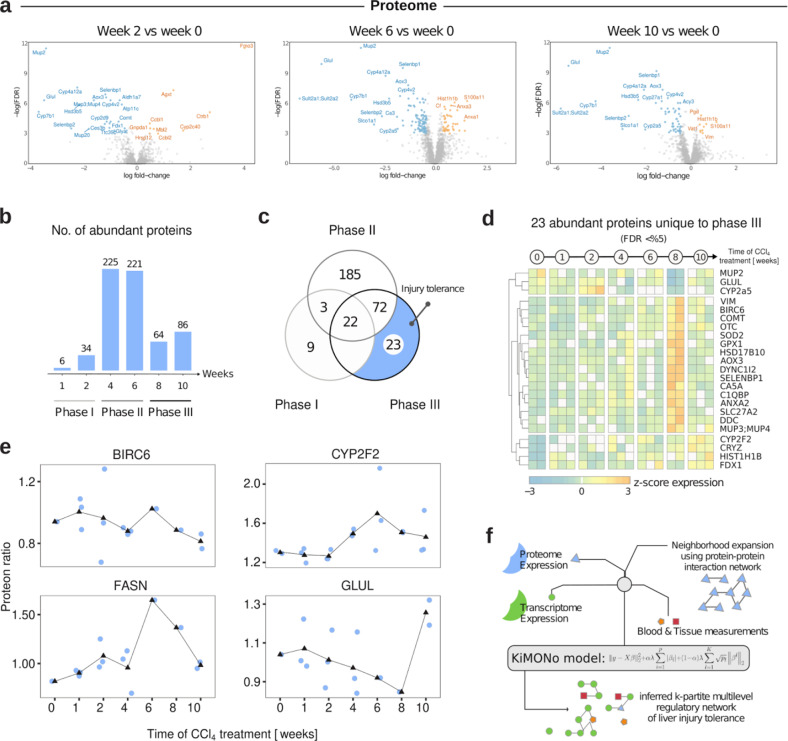
Time-resolved proteomic analysis of liver exposed to CCl_4_. **a** Volcano plots of differentially regulated proteins in weeks two, six, and ten. **b** The number of differentially regulated proteins is visualized via the bar charts. Time points are grouped into three phases characterizing the disease course of liver fibrosis. **c** Venn diagrams illustrate the unique and overlapping amount of proteins between the three phases. **d** Heatmap of uniquely deregulated proteins during phase III (*n* = 2–3 mice per time-point). **e** The longitudinal regulation of the proteins BIRC6, CYP2F2, FASN, and GLUL. **f** KiMONo models performed integration of proteomic, transcriptomic, blood, and tissue measurements.

Proteomic measurements were performed by multiplexing using isobaric labeling with TMT 10-plexes followed by MS/MS analysis, which identified 4222 proteins. For 2278 proteins, expression values were detected in all samples and subjected to bioinformatics and statistical analysis. We derived significant (FDR < 0.05) differentially regulated proteins by comparing control to weeks one, two, four, six, eight, and ten (*n* = 20) (Fig. 3a). Overall we observed similar regulation patterns as in the transcriptomics analysis. We found only six and 34 deregulated proteins in weeks one and two, while 225 and 221 proteins were differentially regulated in weeks four and six and only 64 and 86 in weeks eight and ten (Fig. 3b). The relative number of identified regulated proteins compared to the total number of proteins identified by MS/MS was in line with the observed transcriptomic changes. We identified 22 common deregulated proteins shared between all phases and 23 specific to the tolerance phase (Fig. 3c, d). The pathway enrichment based on proteomic results also showed several enriched metabolic pathways and an induced ECM pathway during phase I and phase II, which is in line with our transcriptomics findings (Supplemental Fig. 3). Time-resolved expression analysis of selected proteins (Fig. 3e) showed a dynamic increase of fibrogenic genes, e.g., collagens, baculoviral IAP repeat-containing protein 6 (BIRC6; anti-apoptotic gene), fatty acid synthase protein (FASN), and stability of metabolic genes like cytochrome P450, family 2, subfamily f, polypeptide 2 (CYP2F2) and glutamine synthetase (GLUL) during the tolerance state. Interestingly, almost all phase I altered genes (95%) and proteins (75%) were also deregulated in the other phases indicating a stereotypical liver response to stress (Figs. 2d and 3d, Heatmaps). Together with the previous results obtained at the different analysis levels, the pattern of differentially regulated genes and proteins suggests a dynamic early liver response in three phases. These were coined: (i) initiation phase (weeks one–two) as immediate response patterns to repeated injuries; (ii) progression phase (weeks four–six) of accumulating molecular changes upon repeated injuries; (iii) tolerance phase (weeks eight–ten) to endure repeated injury.

### Multi-level network analysis of liver tolerance captures human liver disease profiles

**Fig. 4:**
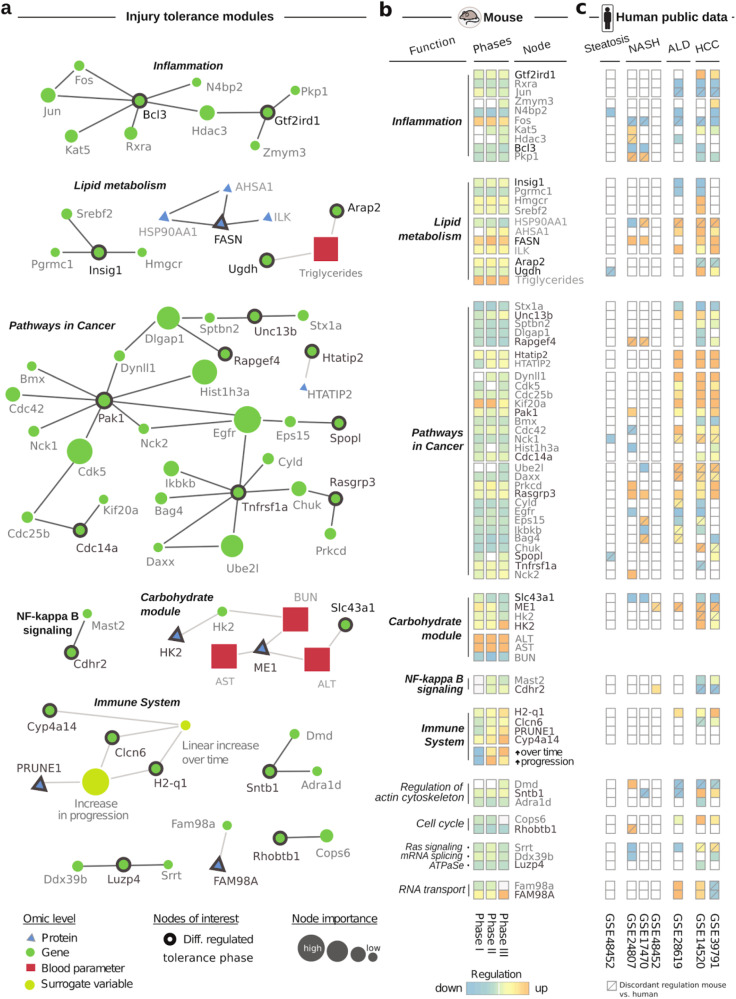
Tolerance specific modules in CCl_4_-induced fibrosis. **a** We identified 13 tolerance phase-specific modules within the multi-omic fibrosis network by extracting differential regulated genes, proteins, and network neighbors. Network nodes are only connected when statistical effects are detected within the data. Node sizes refer to their importance within the network, which relate to the high or low effects of CCl_4_ treatment. **b** Functional annotation and average regulation of network nodes for initiation, progression, and tolerance phase. Significant (FDR < 0.05) downregulation (blue) and upregulation (red) are visualized within the heatmap. Bold node names denote uniquely differential regulation within the tolerance phase. **c** Significantly (FDR < 0.05) differentially expressed genes of seven human studies investigating fatty liver disease (steatosis), non-alcoholic fatty liver disease (NASH), alcoholic liver disease (ALD), and hepatocellular carcinoma (HCC).

To dissect key factors determining the response of the liver to repeated injuries, we generated a fully integrated map combining transcriptomic, proteomic, biochemical, and histopathological data. We incorporated and analyzed all available data using the recently published multi-omic network inference strategy—KiMONo (pipeline Fig. 3f, result Fig. 4a, b) [17]. This analysis strategy allowed us to identify molecular patterns that covary with tissue-level histological parameters. In this inferred multi-level fibrosis network, nodes represent proteins, genes, or biochemical parameters, and connections denote statistically identified effects between them. We trimmed the generated network by excluding all node models with low goodness of fit (R^2 < 0.1) and all effects weaker than beta < 0.002. The filtered network contained 8199 nodes connected via 16398 links. We identified tolerance phase-specific network modules via extracting nodes associated with deregulated unique genes and proteins within the phase. This resulted in 13 distinct network modules consisting of 68 injury-tolerant specific nodes. The minor consists of 2 nodes, while the largest comprises 29 nodes (Fig. 4a, b). On average, functional annotation identified 22.6 significantly (FDR < 0.05) enriched pathways per module. Based on this annotation, we themed the most prominent modules into Inflammation, Lipid metabolism, Pathways in Cancer, Carbohydrate module, NF-kappa B signaling, and Immune system. Even though functions/modules like inflammation have been previously associated with liver fibrosis progression and regression [33], we also detected multi-level evidence for novel mechanisms like lipid metabolism and carbohydrate modules (Fig. 4a, b). These were also the most extensive multi-modal modules and would have been overlooked by only analyzing the different measurements independently. The most extensive module was enriched for Pathways in Cancer, encompassing well-known cancer-related genes orthologous to humans, like Serine/threonine-protein kinase 1 (Pak1), Cell division control protein 42 homolog (Cdc42), or Cyclin-dependent kinase 5 (Cdk5) (Fig. 4a, Supplemental Tables 4 and 5—Module 11) [34–37]. We compared these findings to significant (FDR < 0.05) DE genes of several human liver diseases. DEGs were computed separately for each human disease, thus comparing a gene’s expression to its matched control sample cohort. We identified concordant expression patterns (regulation of human DEG per disease matching mouse direction of regulation) for most of our identified modules, especially with alcoholic liver disease (ALD) and hepatocellular carcinoma (HCC). Within the inflammatory and immune system modules, we found Histocompatibility 2, Q region locus 1 (H2-q1), General transcription factor II-I repeat domain-containing protein 1 (Gtf2ird1), Retinoid X Receptor Alpha (Rxra), Jun Proto-Oncogene, Ap-1 Transcription Factor Subunit (Jun), NEDD4 Binding Protein 2 (N4bp2), and B-cell lymphoma 3-encoded protein (Bcl3) as significantly upregulated genes in human. However, less overlap of significant DE genes was observed between our results, non-alcoholic fatty liver disease (NASH), and steatosis. The profile comparison of lipid metabolism to humans also showed that known key elements like Fasn, Ilk, Ahsa1, Ughd, or Insig1 align with expression profiles from NASH, ALD, and HCC. These findings are also in line with recent literature, which associates these genes with liver diseases [38–41].

### Histological and biochemical validation of multi-level modules identifies lipid metabolism and mild inflammation in tolerance phase

**Fig. 5:**
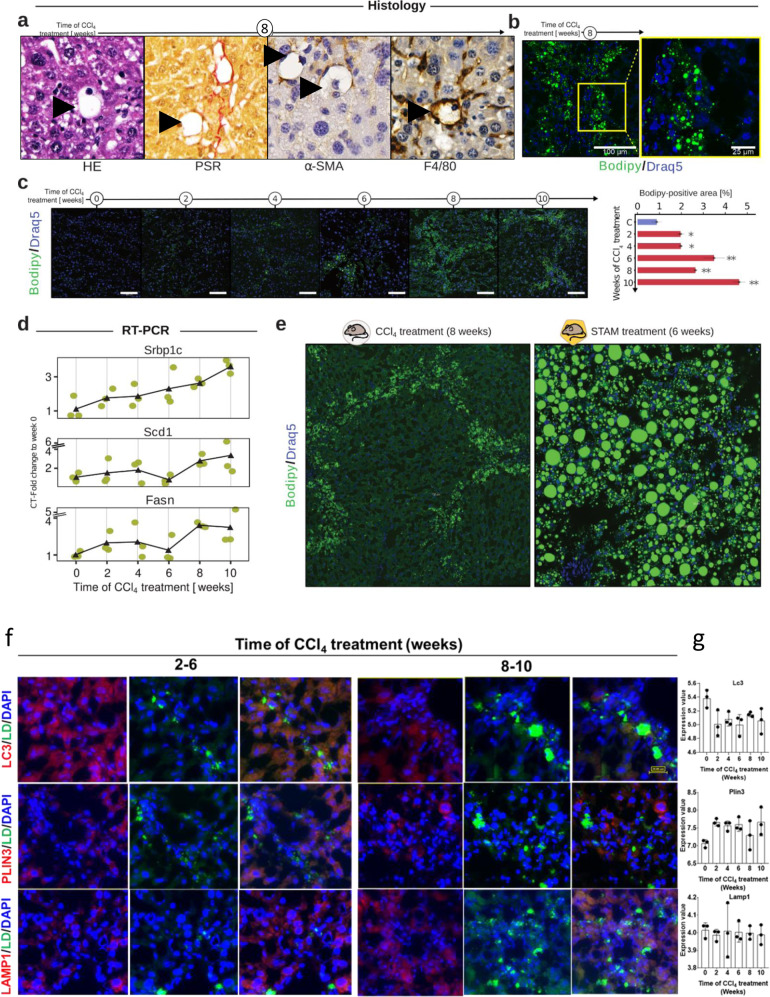
Validation of lipid metabolism induction during the liver response’s tolerance phase. **a** Voids appeared as whitish areas in the liver tissue. The spatial distribution of these voids was along fibrotic regions. HE and PSR staining show the specific circular void structure and, in some cases, small nuclei. Additionally, we found that these voids are surrounded by α-SMA or F4/80 positive cells (arrowhead). **b** Using a specific lipid droplet staining, namely Bodipy, we show that these voids are part of hepatocytes overloaded with lipid droplets. **c** Longitudinal Bodipy staining to visualize and analyze lipid droplet accumulation in a time-resolved manner. Scale bars are 100 µm. **d** mRNA levels validated by RT-PCR of lipid metabolism-related targets Srbp-1c, Scd1, and Fasn. **e** Lower magnification images comparing CCl_4_-induced fibrosis week eight to week six Stellic animal model (steatosis-NASH based model as a positive control for lipid droplet recognition). **f** IF staining of lipid droplets with bodipy (green), microtubule-associated protein 1 A/1B-light (LC3, red), lysosomal-associated membrane protein (LAMP1, red), and prellipin 3 (PLIN3, red). Scale bars are 25 µm. **g** mRNA expression of LC3, PLIN3 and LAMP1 across the different time points.

To validate the detected modules, we first focused on the known association between inflammation and liver disease progression (Supplemental Fig. 4a, b). Histological staining of F4/80 (a marker for resident macrophages) and gene expression (ADGRE1) revealed F4/80 positive cells infiltrated the injured liver tissue, but no further infiltration was observed during the tolerance phase (Supplemental Fig. 4a, b). These results show that we can validate KiMONo’s multi-level modules not only via literature and external data but also experimentally. We focused on the multi-level Lipid metabolism module in a second validation step. This mechanism was identified as being altered explicitly in the tolerance phase. Therefore, we applied immunofluorescence (IF), immunohistochemistry (IHC), and RT-PCR to confirm these findings. Using HE, PSR, ɑ-SMA, and F4/80 staining, we uncovered voids in tissue accumulated within the fibrotic areas (Fig. 5a). By histopathological analysis, we observed that these voids were overloaded with hepatocytes with lipid droplets (LD, Fig. 5b). To investigate intracellular LD further, we established Bodipy staining using cryosections. The results aligned with the multi-level analysis and showed a major increase in intracellular lipid accumulation in a time-dependent manner (Fig. 5c). We underpinned this using quantification of LD (Bodipy positive areas), showing a significant (p < 0.005) lipid accumulation after six weeks, in agreement with the in-silico analysis of KiMONo (Fig. 5c). Next, we validated the gene nodes within the lipid metabolism module via RT-PCR. Here we focused on central regulators of de novo lipogenesis, namely Fasn, sterol regulatory element-binding transcription factor 1 (Srebp-1c), and stearoyl-CoA desaturase 1 (Scd1). These were significantly upregulated within the tolerance phase of CCl_4_-induced fibrosis (Fig. 5d). To further validate that the lipid metabolism is mainly disturbed during the tolerance phase, we analyzed the expression of carnitine palmitoyltransferase 1 (Cpt1), and acyl-CoA oxidase 1 (Acox1), and peroxisome proliferator-activated receptors (PPAR-ɑ and γ). All three showed a downregulation during the initiation and progression phase and recovery to the basal level during the tolerance phase (Supplemental Fig. 5a). Finally, we used a STelic Animal Model (STAM, steatosis-NASH-based mouse model) to compare the size of LD of CCl_4_-tolerated livers as a positive control for LD recognition (Fig. 5e). This comparison shows that LD in CCl_4_-tolerated livers were small compared to the LD inSTAM mice (Fig. 5e). Overall, the validation experiments indicate a tuning down of inflammatory processes during the tolerance phase and confirm the accumulation of tiny LD in CCl_4_-tolerated liver hepatocytes. As a result, these modulations of liver response protect the liver from further CCl_4_-induced damages. LD accumulation is controlled by the balance between lipogenesis and degradation, therefore, we investigated whether lipid degradation is also modulated during initiation or progression stage by auto(lipo)phagy as possible LD degradation mechanism. IF staining (Fig. 5f) and RNA analysis (Fig. 5g) of typical autophagy parameters LC3, PLIN3 and LAMP1 showed no significant alteration between initiation, progression and tolerance phases. The data were in agreement with enrichment plots of autophagy and phagosome components from time-resolved analysis of the array data (Supplemental Fig. 5b). This indicates that de novo synthesis of lipid represents the main factor for droplet accumulation during tolerance phase.

To investigate whether the expression of the 210 identified *tolerance* genes remain altered upon long-term intoxication, we analysed the data of a mouse cohort (GSE167216) that was challenged with CCl_4_ for up to one year [42]. We found 168 shared genes in our dataset (GSE222576) and GSE167216. From these, several displayed similar expression profiles at 2 and 6 months after CCl_4_ exposure (proposed tolerant phase), but showed altered expression profiles at the 12 months time points i.e., among others Hsd17b2, Emcn, Apon, Ugdh, Slc43a1 ggta1, Acss2 Acss3, Abdh4 (Supplemental Fig. 6a, heatmap, red asterisk). Next, to investigate the clinical relevance of identified 210 genes (GSE222576) in CLD cohort of patients (GSE139602; [43] including early chronic liver disease (eCLD, *n* = 5) and compensated cirrhosis (CC, *n* = 8) compared with healthy (*n* = 6) livers (Supplemental Fig. 6b, Venn diagram). Surprisingly, we found that 15 genes (ACACA, COX5B, IL1RN, MMP7, INSIG1, RALGPS1, TSKU, EFHD2, ACSL5, CCDC159, FAM69A, PMVK, SLC35D2, HSDL2, and SPATS2) overlapped with the genes of CC patients, in contrast only 5 genes (S1PR1, PAK1, CLCN6, ACPP and EMCN) overlapped with genes of eCLD patients [43]. In conclusion, our data indicate that the identified and hypothesized tolerance phase upon repeated CCl_4_ intoxication is associated with accumulation of tiny LD and mild inflammation. The tolerance phase starts at around 8 weeks and remains for at least 6 months to cope with repetitive insults and maintain liver function. Our analysis opens a new venue on the presence of gene signatures that are related to compensate against ongoing damage, similarly in mice and patients with liver diseases that could be targeted to interfere with disease progression. Further functional studies are warranted to get further evidence for our hypothesis.

## Discussion

This study identified lipid metabolism and mild inflammation as potent multi-level liver response protection mechanisms against fibrosis. CCl_4_-induced liver fibrosis is a commonly used and highly reproducible model in rodents. CYP2E1 metabolizes CCl_4_ to highly reactive free radical metabolites, particularly trichloromethyl and trichloromethyl peroxy free radicals which attack polyunsaturated fatty acids in membranes causing membrane disruption [44]. We established a fibrosis-induced mouse model with CCl_4_ injections twice a week, for ten weeks. Even though all mice were genetically identical, 10% were unable to cope with acute injury of CCl_4_. This loss might occur due to the inter-individual liver responses, which could be further investigated using single-cell technologies but remains unexplored and unexplained.

Our study design aimed for a matched multi-level data generation to decrease potential biases and variation between the measurements. Therefore, we performed weekly phenotypic, histological, biochemical, and biweekly transcriptomic and proteomic measurements. We applied each measurement technique in the same lobe to reduce the biological variation between samples. Lobe samples were collected once a week over ten weeks. Control samples were collected on weeks one and ten using oil-administered mice. These groups were merged after statistical analysis, which revealed no significant difference between them, increasing the statistical power 2-fold.

The measured levels of circulating liver enzymes ALT and AST indicate a dynamic range of liver response. ALT and AST levels in weeks four and six are in accordance with the previous reports [33]. Also, fibrosis and inflammation features are pronounced at weeks four and six, which was reported before across a similar period (day two & week four) [33].

Independent state-of-the-art proteome and transcriptome analysis was performed, and using PCA, we identified three different liver disease phases: initiation, progression, and tolerance. Statistical power was sufficient to extract significant DE genes in each phase, even though protein and transcriptome data were measured biweekly. However, additional work will be needed to explore how long the tolerance phase progresses after ten weeks of CCl_4_ exposure. Our pathway enrichment analyses detected that ECM and other metabolism-related pathways were active for ten weeks, in line with other recent studies [13]. Noteworthy is that we observe similar pathway activity in our model even though Tuominen et al. [13] used half the dose of CCl_4_ (0.8 g/kg). Also, Knockaert and co-workers observed lipid droplets 3 hours after CCl_4_ administration [45]. Furthermore, in a long-term study (1 year; GSE167216), Ghallab and co-workers also showed that lipid metabolism is induced in CCl_4_-exposed mice [42].

In primary hepatocytes, it is reported that steatotic conditions enhance the inhibition of metabolic enzymes compared to non-steatotic situation [46]. Moreover in human, acetaminophen toxicity is higher in non-obese (BMI: 18.5–24.9 kg/m^2^; 37.5%) than the obese ones (BMI: ≥30 kg/m^2^; 27.5%) [47]. Similar pathways were also confirmed as activated in other model organisms, e.g., rat [48]. This suggests that the observed liver response might be dose, species and strain-independent. Our transcriptomic and proteomic analysis indicates a point where the injured organ cannot cope with excessive ECM accumulation and switches the program to be more tolerant to further damage. However, such complex multi-level liver responses haven’t been covered in other studies [49–51].

We integrated and overlaid evidence at multiple levels, obtaining more profound insights into the liver response regulatory mechanisms. By using our novel knowledge-guided multi-omic network inference tool KiMONo, we integrated all measured information levels in the form of a network. We further extracted 13 modules specific to the tolerance phase and themed them accordingly. Even though the inferred CCl_4_ injury network has the potential to shed light on the initiation phase and progression phase effects as well, we focused on the tolerance phase to identify novel protection mechanisms. This tolerance phase seems to start early after 6 weeks of intoxication (our datasets) and lasts longer than 6 months as shown in Fig. 1 (PSR images), Fig. 4 (pericentral and periportal genes) and Fig. 5 (CYPs expression), by Ghallab et al. [42]. Future work on the initiation and progression phase could lead to insights into first-responder injury processes, like wound healing (ECM accumulation), that are fascinating effects to be further investigated.

The literature validation of single nodes triggered a comparison between our multi-level findings in our murine and human liver disease datasets. Even though we initially obtained 29 different disease data sets, we selected those studies with representative sample sizes. This reduced the amount to seven comprehensive studies on steatosis, NASH, ALD, and HCC. We found several genes that were consistently deregulated in human liver diseases within this data. Although our study has identified high confidence candidate genes for fibrosis tolerance, follow-up with single-cell data and knockdown/overexpression in specific cell types in the liver will be required for functional validation.

So far, we have applied a series of validation experiments for lipid metabolism. All are in line and revealed a significant accumulation of small intracellular lipid droplets during the tolerant phase. At these time points, several lipogenic genes e.g., Srebp1c, Scd1, and Fasn, are significantly induced compared to control and the progression phase. Furthermore, we detected no significantly different regulation of PPAR-α and γ between the initiation, progression, and tolerance phase. This contradicts a previous study that has shown that PPARα is a key protein involved in liver lipid metabolism, and its induction results in de novo hepatic lipogenesis [52]. Next, we can show that several genes were deregulated during compensated liver cirrhosis in human indicating the clinical relevance of our findings.

Moreover, we confirmed sustained inflammatory processes, identified by KiMONo, via staining and detecting F4/80 cells near fibrotic areas. This tuning down of F4/80 expressing cells is due to a fibrosis resolution CCl_4_-based model as reported previously [33]. Therefore, we assume that surviving mice after week six experience a reversion of TG in the blood to an average level, accompanied by increased microvesicular droplets as features of liver tolerance.

**Fig. 6:**
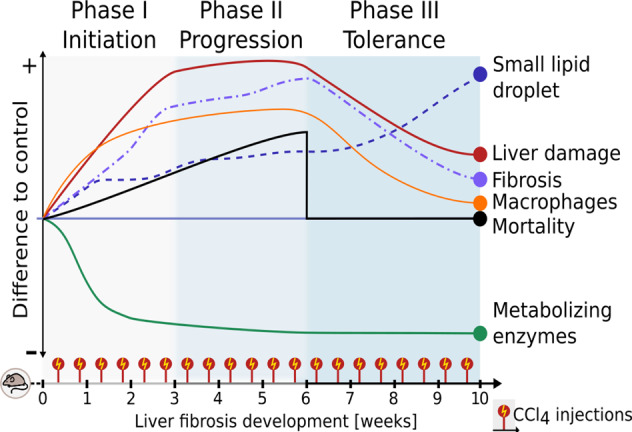
A schematic diagram summarizes liver response to repetitive toxic injuries. In the initiation phase, liver damage, fibrosis, and macrophages were accumulated while down regulating metabolizing enzymes. In the Progression phase, either further accumulation or no change of these parameters compared with the initiation phase. During the tolerance phase, except for dysregulation of lipid metabolism, all parameters had a trend to be normalized. Accumulation of intracellular lipid droplets is a key feature of the tolerant phase and will be studied in the future.

In conclusion, we used a multi-level analysis to identify three phases of early liver response to repeated toxic injuries (Fig. 6). (i) The initiation phase; reflects the first treatment response. Acute liver damage, fibrogenesis, and macrophages accumulation while metabolizing enzymes are downregulated. (ii) The progression phase; characterized by stabilizing all parameters or increasing them slightly. (iii) The tolerance phase; No further mortality is observed. Survivors developed a dysregulation of lipid metabolism while all other parameters were normalized, which led us to hypothesize that the liver has an active auto protection program when harmed repeatedly.

Ultimately, we expect lipid metabolism to be a potential marker for the state of human liver damage under chronic conditions, potentially allowing subsequent studies to approximate the individual point of no return of our otherwise regenerative liver.

## Supplementary information

Supp Materials Cleaned

AJ Checklist

## Data Availability

All data generated or analyzed during this study are available from the corresponding authors upon reasonable request.
