# Supplementary material for: Tolerance of repeated toxic injuries of murine livers is associated with steatosis and inflammation
Source: Cell Death Dis. 2023 Jul 12;14(7):414. doi: 10.1038/s41419-023-05855-4 (PMC10338629; doi:10.1038/s41419-023-05855-4)
Supplement: Supplementary file 1 — Supp Materials Cleaned [file 41419_2023_5855_MOESM1_ESM.docx]

## **Supplemental Materials**

# **Tolerance of repeated toxic injuries of murine livers is associated with steatosis and inflammation**

Seddik Hammad^1,2*#^, Christoph Ogris^3#^, Amnah Othman^4^, Pia Erdoesi^1^, Wolfgang Schmidt-Heck^5^, Ina Biermayer^6^, Barbara Helm^6^, Yan Gao^1^, Weronika Piorońska^1^, Christian Holland^4^, Lorenza A. D'Alessandro^6^, Carolina de la Torre^7^, Carsten Sticht^7^, Sherin Al Aoua^8^, Fabian J. Theis^3^, Heike Bantel^8^, Matthias P. Ebert^9,10,11^, Ursula Klingmüller^6^, Jan G. Hengstler^4^, Steven Dooley^1#^, Nikola S. Mueller^3*#^

^1^Molecular Hepatology Section, University Medical Center Mannheim, Medical Faculty Mannheim, Heidelberg University, 68167-Mannheim, Germany

^2^Department of Forensic Medicine and Veterinary Toxicology, Faculty of Veterinary Medicine, South Valley University, 83523-Qena, Egypt

^3^Institute of Computational Biology, Helmholtz-Zentrum München, German Research Center for Environmental Health, 85764-Neuherberg, Germany

^4^Department of Toxicology, Leibniz Research Centre for Working Environment and Human Factors (IfADo), 44139-Dortmund, Germany

^5^Leibniz Institute for Natural Product Research and Infection Biology - Hans Knoell Institute, 07745-Jena, Germany

^6^Division Systems Biology of Signal Transduction, German Cancer Research Center (DKFZ), INF 280, 69120-Heidelberg, Germany

^7^Core Facility Next Generation Sequencing, Medical Faculty Mannheim, Heidelberg University, Mannheim, Germany.

^8^Department of Gastroenterology, Hepatology and Endocrinology, Hannover Medical School, Carl-Neuberg-Strasse 1, 30625 Hannover, Germany^9^Department of Medicine II, University Medical Center Mannheim, Medical Faculty Mannheim, Heidelberg University, 68167-Mannheim, Germany

^10^Mannheim Institute for Innate Immunoscience (MI3), University Medical Center Mannheim, Medical Faculty Mannheim, Heidelberg University, 68167-Mannheim, Germany

^11^Clinical Cooperation Unit Healthy Metabolism, Center of Preventive Medicine and Digital Health, University Medical Center Mannheim, Medical Faculty Mannheim, Heidelberg University, 68167-Mannheim, Germany

^#^Equally contributing

*Corresponding authors:

**Dr. Seddik Hammad.** Molecular Hepatology Section. Department of Medicine II. University Medical Center Mannheim. Medical Faculty Mannheim. Heidelberg University. 68167-Mannheim. Germany. Email: [seddik.hammad@medma.uni-heidelberg.de](mailto:seddik.hammad@medma.uni-heidelberg.de) Tel: 0049 0621 383 5603

**Dr. Nikola Müller.** Institute of Computational Biology. Helmholtz Zentrum München. Ingolstädter Landstr. 1. 85764-Neuherberg. Email: nikola.mueller[@helmholtz-muenchen.de](mailto:christoph.ogris@helmholtz-muenchen.de)


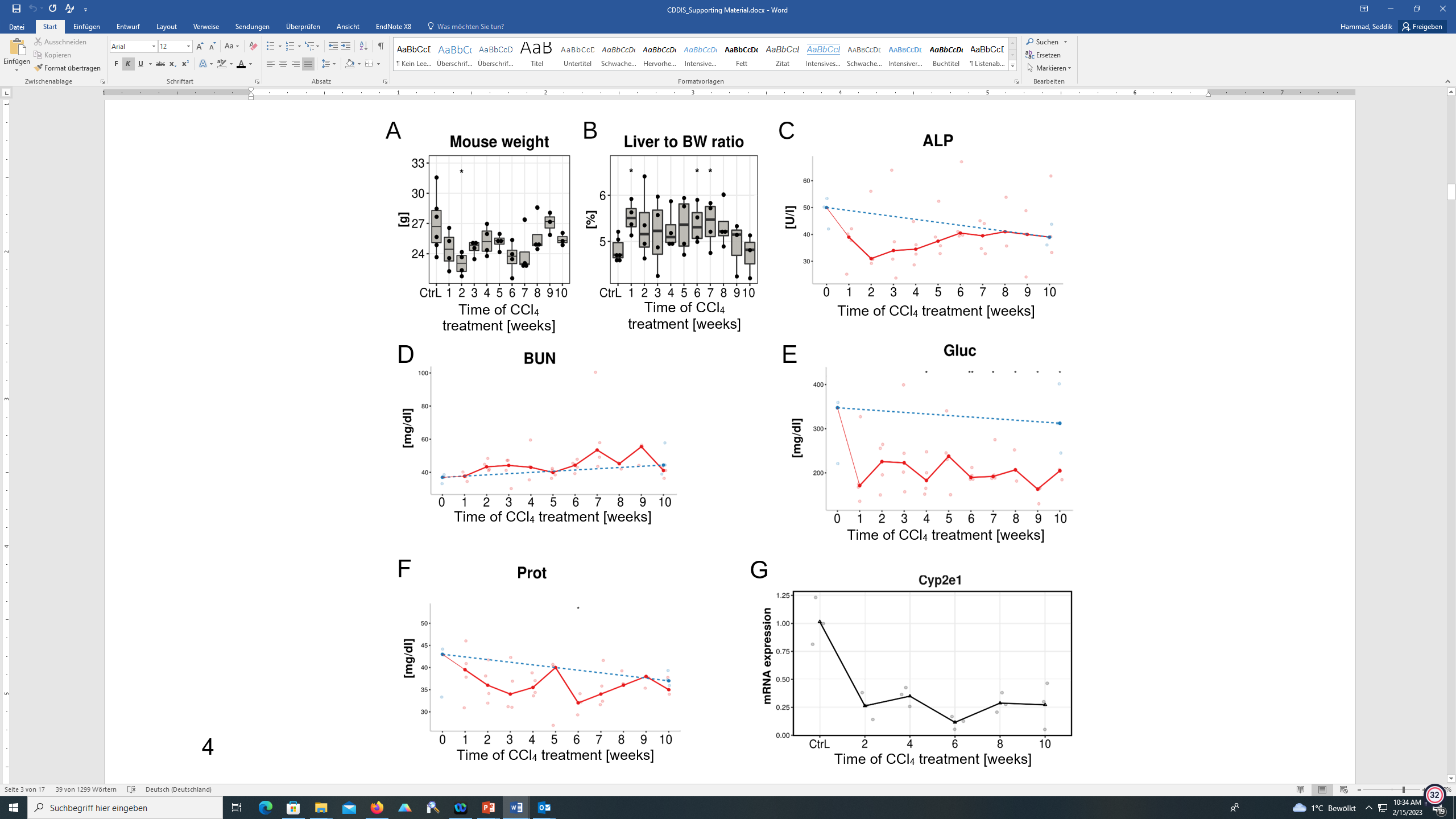
**Supplemental Figure 1: Mouse and liver weight and metabolic parameters during fibrogenesis.** (a) Mouse weight. (b) Liver weight as a ratio of body weight. (c-f) Time resolved measurements of ALP, BUN Gluc, and total protein levels in blood of mice exposed to CCl_4_. (g) mRNA level of Cyp2e1 analyzed by RT-PCR. Results are shown as mean ± SD, and were compared by two-way ANOVA test. *p <0.05 compared to 0 week (control). n=3-6 per group. CCl_4_, carbon tetrachloride; ALP, alkaline phosphatase; BUN, blood urea nitrogen; Gluc, glucose; Prot, total protein; Cyp2e1, Cytochrome P4502e1.


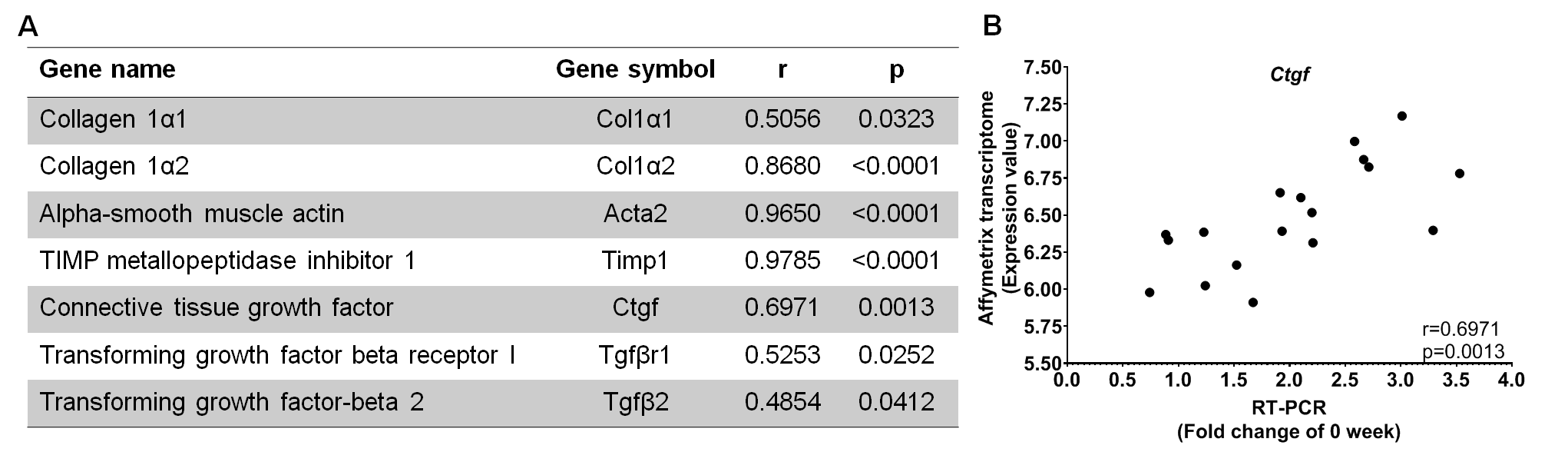
**Supplemental Figure 2:** Analysis of 7 selected reference genes comparing RT-PCR and Affymetrix-based transcriptome in 18 mice. (a) Table shows Coefficient of Pearson correlation (r) and p values between each target analysed by RT-PCR and transcriptomics. (b) Connective tissue growth factor (Ctgf) result is blotted as an example of correlation between fold change of RT-PCR of 0 week and expression value based on transcriptome.


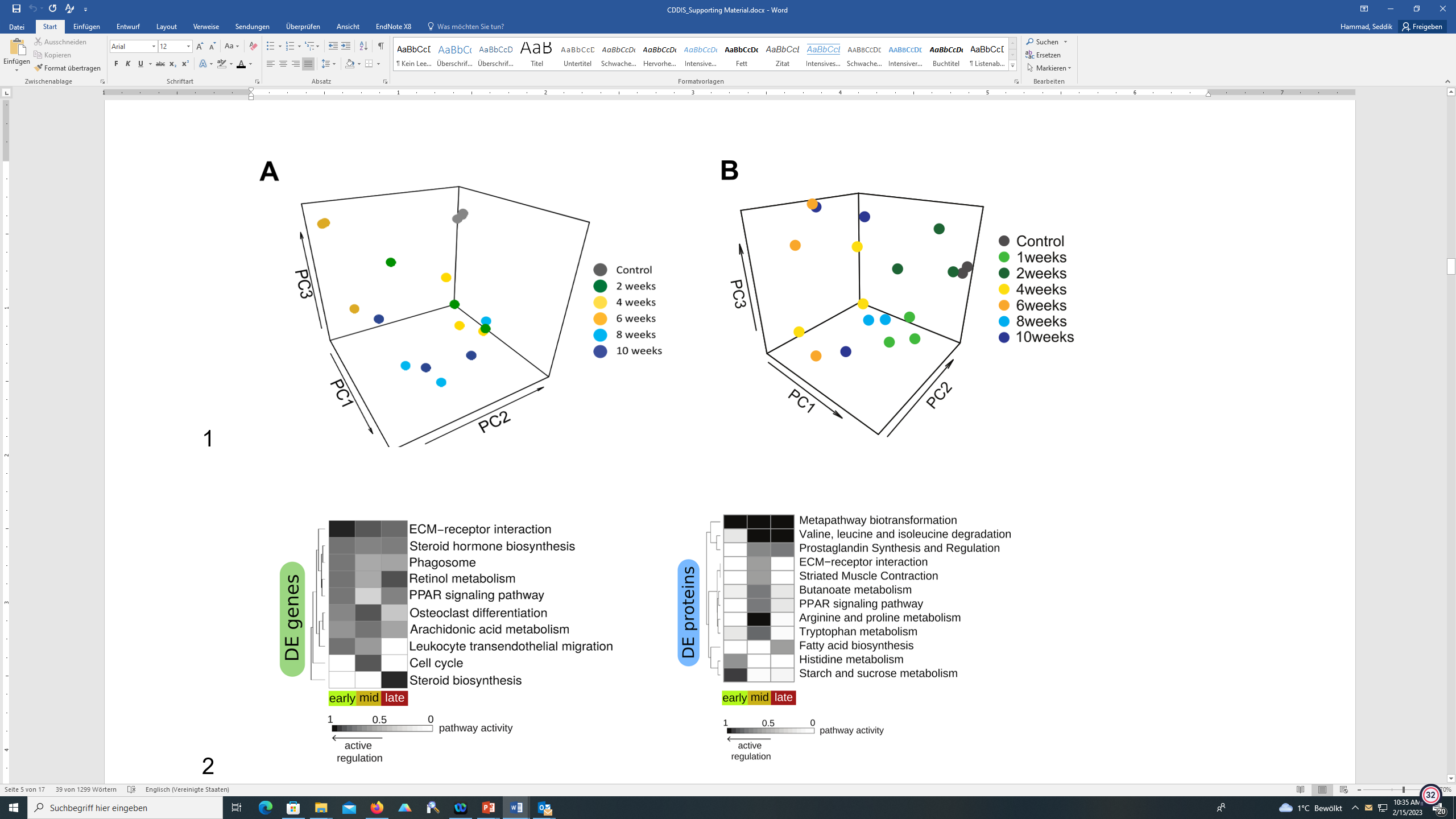
**Supplemental Figure 3:** (a) PCA of the transcriptome across all time points. (b) PCA of the proteome across all time points. Pathway analysis of DE genes and abundant proteins across all three identified phases upon chronic CCl_4_ injections.

**
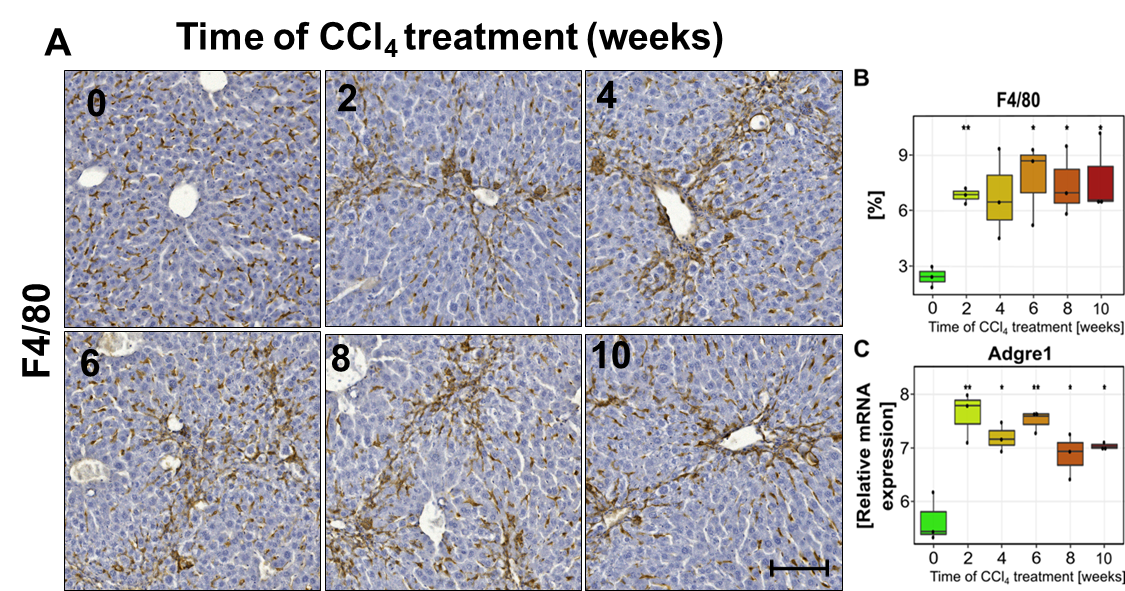
**

**Supplemental 4. F4/80 expression is sustained during the adaptation phase after repeated CCl_4_ injections. (**a) Liver sections were prepared from liver tissue samples of control and mice with CCl_4_ treatment for 10 weeks and subjected to immunostaining against F4/80. Scale bars are 100µm and 200µm for closeups and overview images, respectively. (b) Quantification of positive F4/80 signals from stained slides over the total area. (c) mRNA expression of F4/80 assigned gene (Adgre1) extracted from transcriptomic datasets. Results were expressed as mean ± SD, and were compared by two-way ANOVA test. *p <0.05, **p <0.01 compared to 0 week (control). n=3-6 per group.


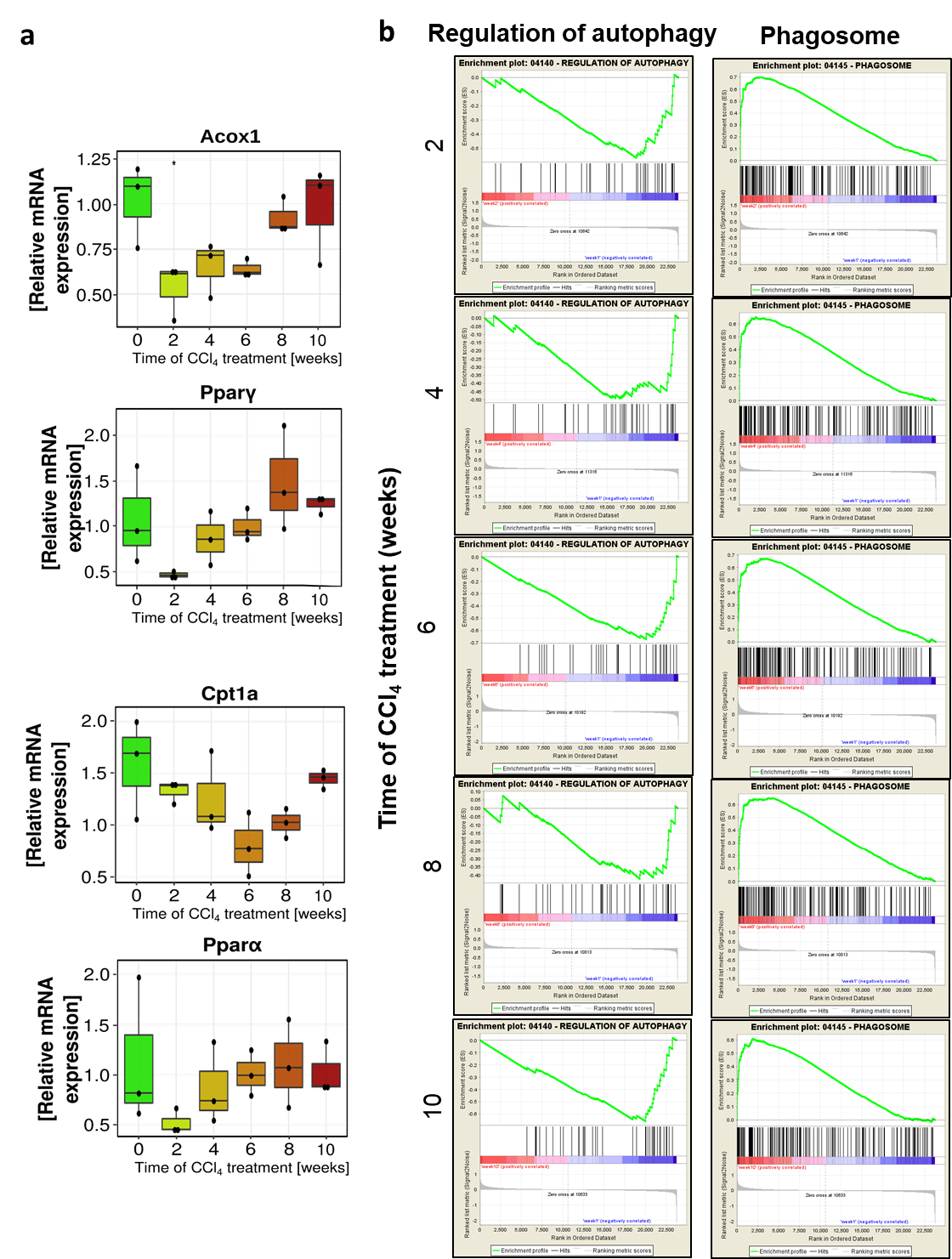
**Supplemental Figure 5: Bioinformatics analysis of lipid metabolism related targets and pathways. a)** mRNA levels of lipid metabolism-related genes i.e. Acox1, Cpt1a, Ppar𝞬 and Pparɑ are analysed by RT-PCR . Results are expressed as mean ± SD, and were compared by two-way ANOVA test. *p <0.05 compared to 0 week (control). n=3-6 per group. (b) Enrichment plot for autophagy and phagosome in a time-resolved experiment from transcriptomics datasets (GSE222576) indicating no clear difference between initiation, progression and tolerance phases after CCl_4_ intoxication.


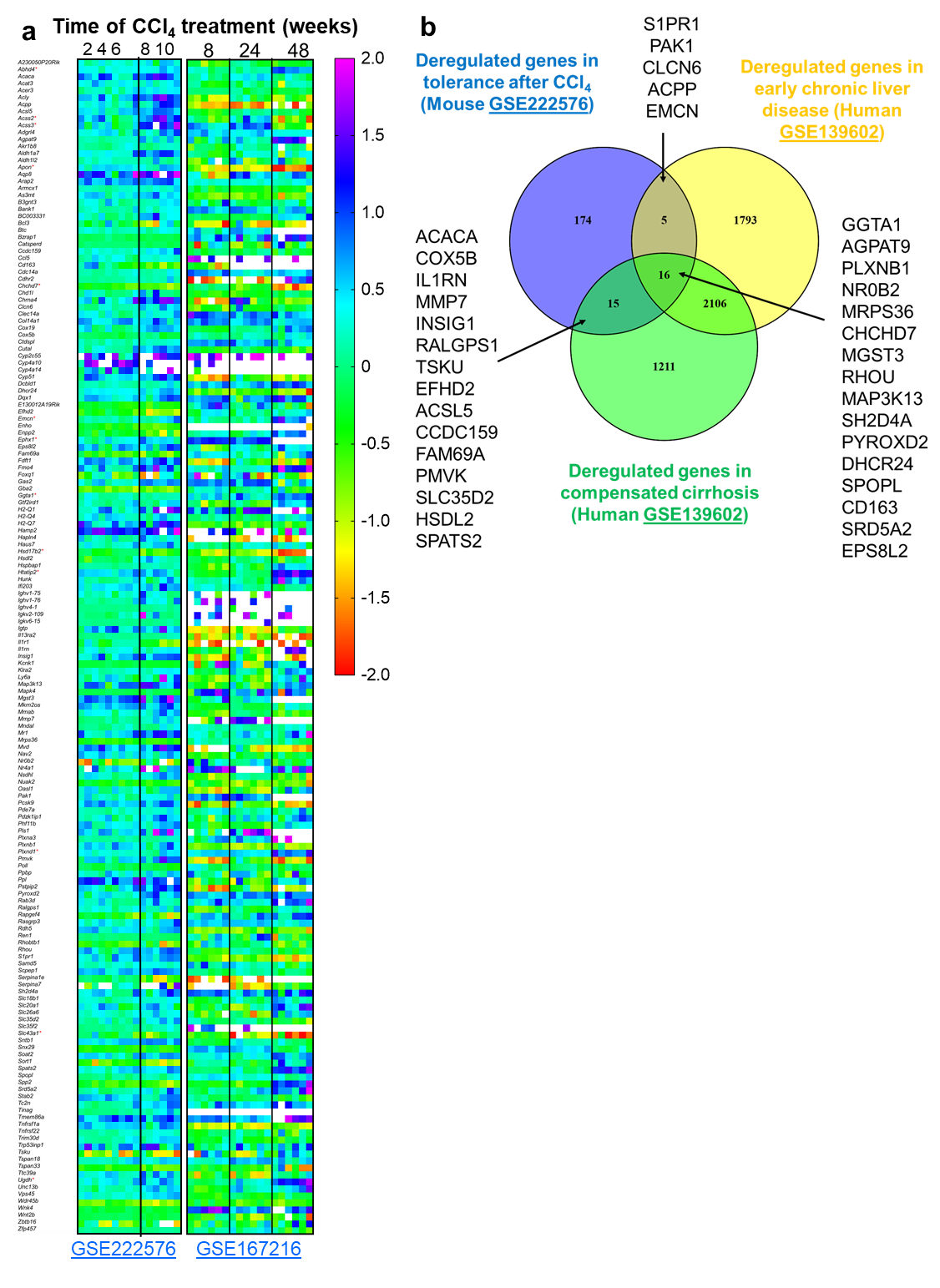


**Supplemental Figure 6: Analysis of long-term CCl_4_ intoxicated mice and human cohort of liver patients.** a) Heatmap shows the overlapped 168 genes between GSE222576 and long-term CCl_4_ intoxicated mice (GSE167216) from Ghallab et al (42). Asterisk refers to exemplary genes that remain during the disease course and changed after 48 weeks of CCl_4_ treatment. b) Comparative analysis of identified 210 targets from mouse analysis and early chronic liver disease (eCLD) and compensated cirrhosis (CC) patients (GSE139602). We found 15 genes were consistently deregulated in tolerance phase and CC patients.

## **Supplemental Tables**

**Supplemental Table 1:** RNA concentration and integrity isolated from oil and CCl_4_-exposed mouse livers. This RNA was used for transcriptomics and RT-PCR analysis. 50µl was prepared (100ng/µl) by dilution in nuclease-free water Affymetrix (J71786 Water, Thermo Scientific). For Affymetrix transcriptomics 100 ng/µl (20µl) was used.


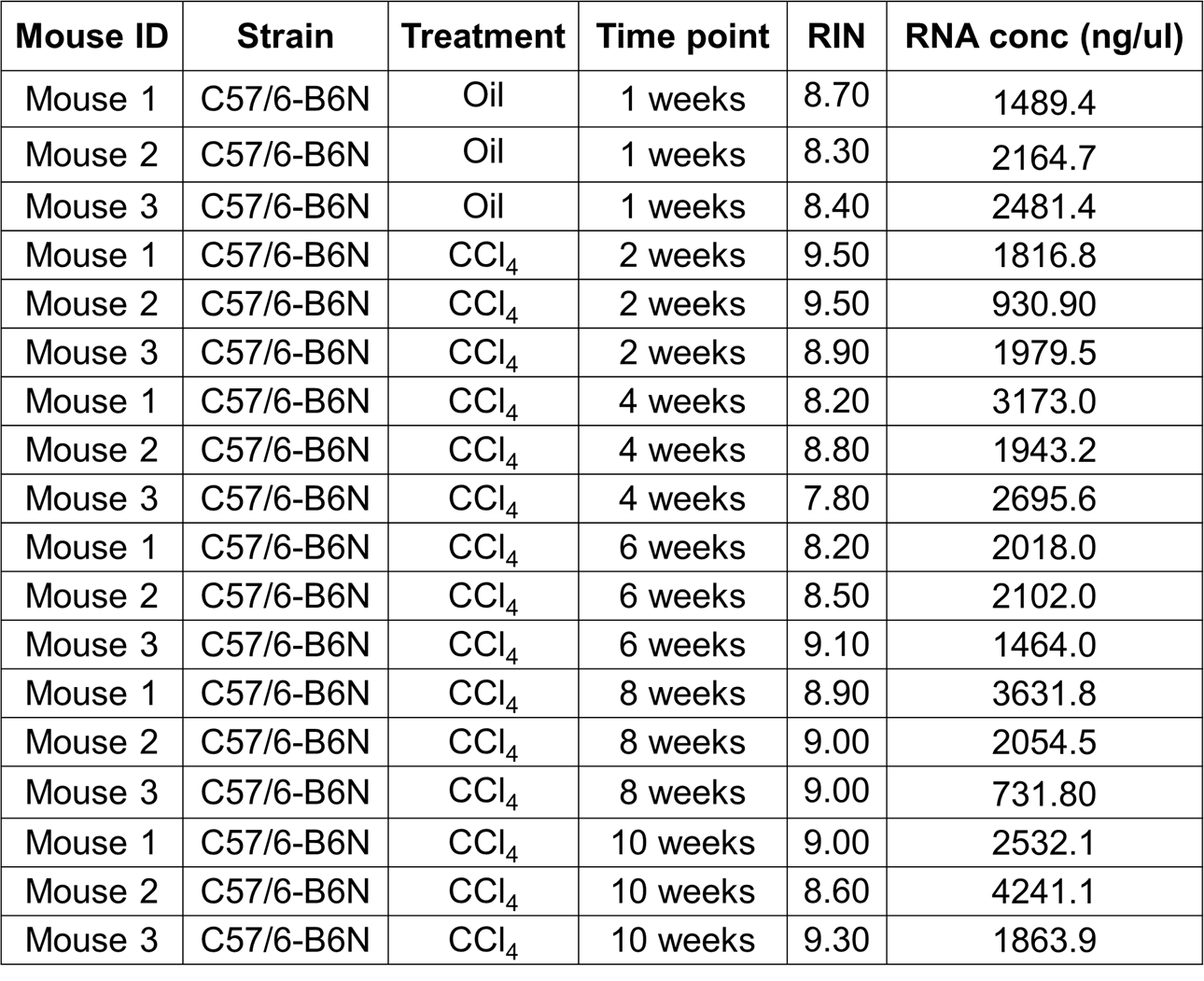


**Supplemental Table 2**. Sequences of primer pairs used for real time PCR.

| **Gene** | **Forward** | **Reverse** |
| --- | --- | --- |
| Acox1 | GAATTTGGCATCGCAGACCC | CGGGTGCATCCATTTCTCCT |
| Acta2 | TTCGCTGTCTACCTTCCAGC | GAGGCGCTGATCCACAAAAC |
| Col1α1 | GGAGAGAGCATGACCGATGG | AAGTTCCGGTGTGACTCGTG |
| Col1α2 | AGTCGATGGCTGCTCCAAAA | AGCACCACCAATGTCCAG AG |
| Cpt1a | CGGACTCCGCTCGCTCATT | GGGAGGGGTCCACTTTGGTA |
| Ctgf | AGATTGGAGTGTGCACTGCCAAAG | TCCAGGCAAGTGCATTGGTATTTG |
| Cyp2e1 | CGTTGCCTTGCTTGTCTGGA | AAGAAAGGAATTGGGAAAGGTCC |
| Fasn | ACAATGGACCCCCAGCTTCG | CAGACGCCAGTGTTCGTTCC |
| Pparα | TGCAGCCTCAGCCAAGTTGAA | GTTCCCGAACTTGACCAGCC |
| Pparγ | ACGTTCTGACAGGACTGTGT | CTGTGTCAACCATGGTAATTTCA |
| Ppia | GAGCTGTTTGCAGACAAAGTC | CCCTGGCACATGAATCCTGG |
| Scd1 | AACAGTGCCGCGCATCTCTA | GAAGCCCAAAGCTCAGCTACTC |
| Srbp1c | GGAGCCATGGATTGCACATT | GGCCCGGGAAGTCACTGT |
| Tgfβ2 | GCAGATCCTGAGCAAGCTG | GTAGGGTCTGTAGAAAGTGG |
| Tgf-βr1 | GAACTGTTTTGATTGGCATC | AAGAAGGGACCTACACTATTT |
| Timp1 | GGCATCTGGCATCCTCTTGT | ACTCTTCACTGCGGTTCTGG |

**Supplemental Table 3.** Publically available Human GEO datasets.


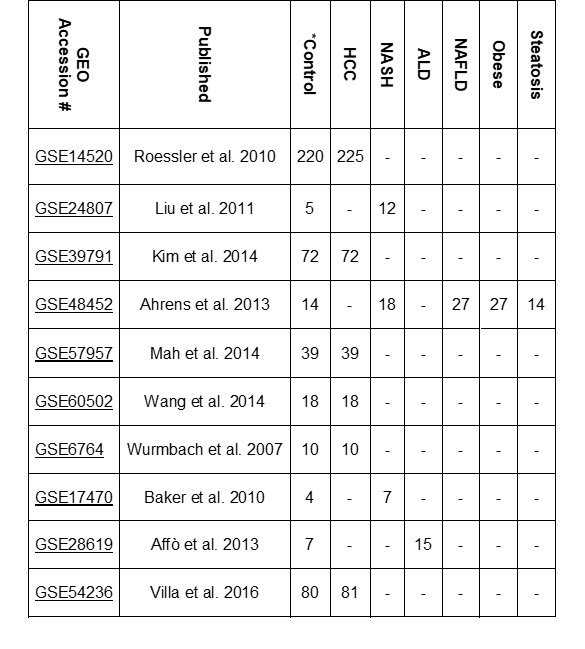


**Supplemental Table 4:** Network nodes representing mouse genes, and proteins, their subnetwork and the corresponding human ortholog.

| **Mus Musculus Node** | **Subnetwork** | **Homo Sapiens Ortholog** |
| --- | --- | --- |
| COPS6 | 1 | COPS6 |
| RHOBTB1 | 1 | RHOBTB1 |
| HTATIP2 | 2 | HTATIP2 |
| Q9Z2G9 | 2 | HTATIP2 |
| CDHR2 | 3 | CDHR2 |
| MAST2 | 3 | MAST2 |
| ARAP2 | 4 | ARAP2 |
| SNHG14 | 4 |  |
| UGDH | 4 | UGDH |
| CLCN6 | 5 | CLCN6 |
| CYP4A14 | 5 |  |
| H2-Q1 | 5 | HLA-A,HLA-B,HLA-C,HLA-D,HLA-E,HLA-F |
| Q8BIW1 | 5 | PRUNE1 |
| ADRA1D | 6 | ADRA1D |
| DMD | 6 | DMD |
| SNTB1 | 6 | SNTB1 |
| BCL3 | 7 | BCL3 |
| FOS | 7 | FOS |
| GTF2IRD1 | 7 | GTF2IRD1 |
| HDAC3 | 7 | HDAC3 |
| JUN | 7 | JUN |
| KAT5 | 7 | KAT5 |
| N4BP2 | 7 | N4BP2 |
| PKP1 | 7 | PKP1 |
| RXRA | 7 | RXRA |
| ZMYM3 | 7 | ZMYM3 |
| DX39B | 8 |  |
| LUZP4 | 8 | LUZP4 |
| SRRT | 8 | SRRT |
| HMGCR | 9 | HMGCR |
| INSIG1 | 9 | INSIG1 |
| PGRMC1 | 9 | PGRMC1 |
| SREBF2 | 9 | SREBF2 |
| O55222 | 10 | ILK |
| P07901 | 10 | HSP90AA1 |
| P19096 | 10 | FASN |
| Q8BK64 | 10 | AHSA1 |
| BAG4 | 11 | BAG4 |
| BMX | 11 | BMX |
| CDC14A | 11 | CDC14A |
| CDC25B | 11 | CDC25B |
| CDC42 | 11 | CDC42 |
| CDK5 | 11 | CDK5 |
| CHUK | 11 | CHUK |
| CYLD | 11 | CYLD |
| DAXX | 11 | DAXX |
| DLGAP1 | 11 | DLGAP1 |
| DYNLL1 | 11 | DYNLL1 |
| EGFR | 11 | EGFR |
| EPS15 | 11 | EPS15 |
| IKBKB | 11 | IKBKB |
| KIF20A | 11 | KIF20A |
| NCK1 | 11 | NCK1 |
| NCK2 | 11 | NCK2 |
| PAK1 | 11 | PAK1 |
| PRKCD | 11 | PRKCD |
| RAPGEF4 | 11 | RAPGEF4 |
| RASGRP3 | 11 | RASGRP3 |
| SPOPL | 11 | SPOPL |
| SPTBN2 | 11 | SPTBN2 |
| STX1A | 11 | STX1A |
| TNFRSF1A | 11 | TNFRSF1A |
| UBE2I | 11 | UBE2I |
| UNC13B | 11 | UNC13B |
| FAM98A | 12 | FAM98A |
| Q3TJZ6 | 12 | FAM98A |

**Supplemental Table 5.** Pathway annotation results of all 13 distinct modules.

## <https://docs.google.com/spreadsheets/d/1FJSHmeTYpMRkU4UmHRfPpuJCrZybnrELAGHbwhodYNI/edit?usp=sharing>

## **SUPPLEMENTAL REFERENCES**

Roessler S, Jia HL, Budhu A, Forgues M et al. A unique metastasis gene signature enables prediction of tumor relapse in early-stage hepatocellular carcinoma patients. Cancer Res 2010 Dec 15;70(24):10202-12. PMID: 21159642

Liu W, Baker SS, Baker RD, Nowak NJ et al. Upregulation of hemoglobin expression by oxidative stress in hepatocytes and its implication in nonalcoholic steatohepatitis. PLoS One 2011;6(9):e24363. PMID: 21931690

Kim JH, Sohn BH, Lee HS, Kim SB et al. Genomic predictors for recurrence patterns of hepatocellular carcinoma: model derivation and validation. PLoS Med 2014 Dec;11(12):e1001770. PMID: [25536056](https://www.ncbi.nlm.nih.gov/pubmed/25536056)

Ahrens M, Ammerpohl O, von Schönfels W, Kolarova J et al. DNA methylation analysis in nonalcoholic fatty liver disease suggests distinct disease-specific and remodeling signatures after bariatric surgery. Cell Metab 2013 Aug 6;18(2):296-302. PMID: [23931760](https://www.ncbi.nlm.nih.gov/pubmed/23931760)

Mah WC, Thurnherr T, Chow PK, Chung AY et al. Methylation profiles reveal distinct subgroup of hepatocellular carcinoma patients with poor prognosis. PLoS One 2014;9(8):e104158. PMID: [25093504](https://www.ncbi.nlm.nih.gov/pubmed/25093504)

Wang YH, Cheng TY, Chen TY, Chang KM et al. Plasmalemmal Vesicle Associated Protein (PLVAP) as a therapeutic target for treatment of hepatocellular carcinoma. BMC Cancer 2014 Nov 6;14:815. PMID: [25376302](https://www.ncbi.nlm.nih.gov/pubmed/25376302)

Wurmbach E, Chen YB, Khitrov G, Zhang W et al. Genome-wide molecular profiles of HCV-induced dysplasia and hepatocellular carcinoma. Hepatology 2007 Apr;45(4):938-47. PMID: [17393520](https://www.ncbi.nlm.nih.gov/pubmed/17393520)

Baker SS, Baker RD, Liu W, Nowak NJ et al. Role of alcohol metabolism in non-alcoholic steatohepatitis. PLoS One 2010 Mar 8;5(3):e9570. PMID: [20221393](https://www.ncbi.nlm.nih.gov/pubmed/20221393)

Affò S, Dominguez M, Lozano JJ, Sancho-Bru P et al. Transcriptome analysis identifies TNF superfamily receptors as potential therapeutic targets in alcoholic hepatitis. Gut 2013 Mar;62(3):452-60. PMID: [22637703](https://www.ncbi.nlm.nih.gov/pubmed/22637703)

Villa E, Critelli R, Lei B, Marzocchi G et al. Neoangiogenesis-related genes are hallmarks of fast-growing hepatocellular carcinomas and worst survival. Results from a prospective study. Gut 2016 May;65(5):861-9. PMID: [25666192](https://www.ncbi.nlm.nih.gov/pubmed/25666192)
